# Case Report: A Novel Homozygous Variant of the *SERPINF1* Gene in Rare Osteogenesis Imperfecta Type VI

**DOI:** 10.3390/ijms24076672

**Published:** 2023-04-03

**Authors:** Irina Zh. Zhalsanova, Anna Evgenievna Postrigan, Nail Raushanovich Valiakhmetov, Nikita Aleksandrovich Kolesnikov, Daria Ivanovna Zhigalina, Aleksei Andreevich Zarubin, Valeria Viktorovna Petrova, Larisa Ivanovna Minaycheva, Gulnara Narimanovna Seitova, Nikolay Alekseevich Skryabin, Vadim Anatolevich Stepanov

**Affiliations:** Research Institute of Medical Genetics, Tomsk National Research Medical Center, Tomsk 634050, Russia; irina.zhalsanova@medgenetics.ru (I.Z.Z.);

**Keywords:** osteogenesis imperfecta, connective tissue disorders, *SERPINF1*, next-generation sequencing

## Abstract

Osteogenesis imperfecta (OI) is a group of connective tissue disorders with different types of inheritance. OI is characterized by bone fragility and deformities, frequent fractures, low bone-mineral density, and impaired bone micro-architectonics. We described here a case of a one-year-old Tuvan patient with multiple fractures. The disease manifestation occurred first at 12 weeks of age as a shoulder joint bruise, and during the year, the patient sustained 27 fractures. Genetic testing revealed a novel homozygous mutation, c.259_260insCGGCC (p.T87fs), in the *SERPINF1* gene. This insertion leads to an open-reading frameshift, and the mutation is not represented in the databases. Mutations in *SERPINF1* lead to type VI OI, the clinical picture of which is similar to the disease phenotype manifestation of the patient. Thus, the patient’s diagnosis was established by finding a novel pathogenic variant in the *SERPINF1* gene.

## 1. Introduction

Osteogenesis imperfecta (OI) is a heterogeneous group of diseases with different types of inheritance. These diseases are characterized by low bone mass and bone fragility, causing significant complications due to pain, immobility, skeletal deformities, and growth deficiency. The OI clinical symptoms vary from one to two fractures in a lifetime to obvious skeletal anomalies that can lead to death. In addition to bone disorders, patients have dentin anomalies, gray-blue sclera, hearing loss, muscle weakness, and disorders in the cardiovascular and respiratory systems [1]. The severity of the disease depends on the frequency of fractures, progressive deformity, and loss of mobility. The disease can manifest both during pregnancy and later in life.

According to the Sillence classification, osteogenesis imperfecta is divided into four main types depending on the patient’s clinical and radiological data (I–IV) [2]. When F.H. Glorieux presented an osteogenesis imperfecta classification, he added four more types of OI (V, VI, VII, VIII) which were not associated with the pathology of type I collagen [2]. So far, 18 forms of OI have been described depending on the target gene and whether it is inherited in an autosomal dominant, autosomal recessive, or X-linked recessive manner. According to the OMIM database, there are 22 osteogenesis imperfecta types (166200; 166210; 259420; 166220; 610967; 613982; 610682; 610915; 259440; 613848; 610968; 613849; 614856; 615066; 615220; 616229; 616507; 617952; 301014; 618644; 619131; 619795). I–IV OI types have an autosomal dominant type of inheritance and are caused in 85–90% of cases by mutations in the genes encoding type I collagen—*COL1A1* and *COL1A2* [3]. The remaining cases are much less common and are caused by pathogenic variants of non-collagenous genes. These genes code for proteins involved in collagen biosynthesis or pathways associated with differentiation and bone cell mineralization (*IFITM5, SERPINF1, CRTAP, P3H1, PPIB, SERPINH1, FKBP10, SP7, BMP1, TMEM38B, WNT1, CREB3L1, SPARC, TENT5A, MBTPS2, MESD, KDELR2, CCDC134*).

In the present work, we describe a new homozygous mutation in the *SERPINF1* gene in a Tuvan child with osteogenesis imperfecta type VI.

## 2. Patient Report

In November 2021, a family with a one-year-old boy with multiple fractures came to the inpatient department of the Tomsk National Research Medical Center (TNRMC) genetics clinic. According to the proband parents, they are not relatives and come from different settlements. The family belongs to the Tuva nationality. The proband parents are clinically healthy. The child was the third child born from a total of four pregnancies. The child was born from the pregnancy presented with mild toxicosis and fetal hypoxia. The birth weight and length were 3422 g and 50 cm, respectively, with an Apgar score of 7/8. The proband’s mother has a history of one spontaneous abortion at 10 weeks. One brother, who is seven years older, has unspecified epilepsy, while the five-year-old sister is healthy. The disease manifestation occurred at three months of age with a bruise of the shoulder joint. The first closed displaced fracture of the right femur occurred at seven months of age. The patient developed a closed fracture of the tibial shaft lower third and a closed displaced fracture of the left humerus at nine months of age. The patient had fractures of the 8–9–10 ribs on the right and a compression fracture of the vertebrae L1–L3 by the age of twelve months old.

During the patient’s initial examination in the TNRMC genetic clinic at 1.2 years of age, the patient’s mother noted immobility of the right upper limb that had lasted for one week. On examination, the doctors noted the child’s anxiety. From traumatologist and X-ray examinations, a non-displaced closed fracture was revealed in the upper third of the patient’s right humerus, and a closed non-displaced consolidating fracture of the right radius in the proximal section was also detected. We diagnosed a general condition of moderate severity due to the following movement disorders. The patient had diffuse muscular hypotension and did not sit, stand, or walk. When the right upper limb was brought to the body, there were no active movements in the joints. A complete blood count revealed leukocytosis—16.9 × 10^9^, lymphocytosis—65.8%, thrombocytosis—437 × 10^9^, and significantly increased ESR (erythrocyte sedimentation rate)—41 mm/h. Biochemical studies showed the normal level of alkaline phosphatase—447 U/L and calcium—2.58 mmol/L and an increase in C-reactive protein (6 mg/L). Calcium in the urine was reduced—0.47 mmol/day. A search for the genetic cause of the phenotype was recommended. As of March 2023, the patient has 27 fractures; the patient is not transportable. At the moment, the patient is receiving treatment with zoledronic acid and vitamin D in age-appropriate dosages. This treatment started last year, and the patient’s mother noted an improvement.

## 3. Results

### 3.1. Massively Parallel Sequencing

The patient was sent for target panel sequencing, including the genes *COL1A1* and *COL1A2*. We have not found pathogenic mutations in these genes. The next step was whole-exome sequencing. The whole-exome sequencing of the patient’s DNA revealed a c.259_260insCGGCC (p.T87fs) variant in the *SERPINF1* gene in a homozygous state.

**Figure 1:**
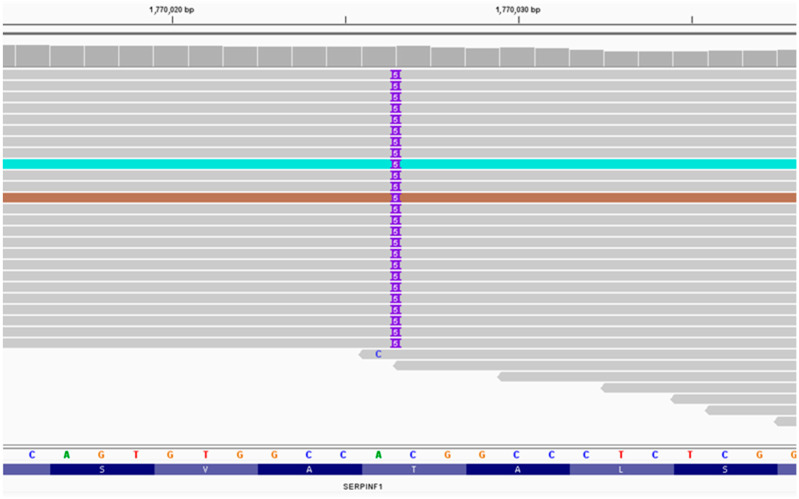
NGS sequencing (IGV browser).

We found a nucleotide sequence frameshift insertion (5 nucleotide duplications) variant in exon 3 of the *SERPINF1* gene (chr17:1,770,026A>ACGGCC) (Figure 1). The variant was not previously described in databases. The variant results in the replacement of threonine at position 84, which leads to the premature formation of a stop codon and impairs protein synthesis (p.T87fs; NM_001329903). This nucleotide sequence variant was not registered in the gnomAD database. Thus, according to the ACMG (The American College of Medical Genetics and Genomics) criteria, this variant of the nucleotide sequence is regarded as pathogenic.

### 3.2. Sanger Sequencing

**Figure 2:**
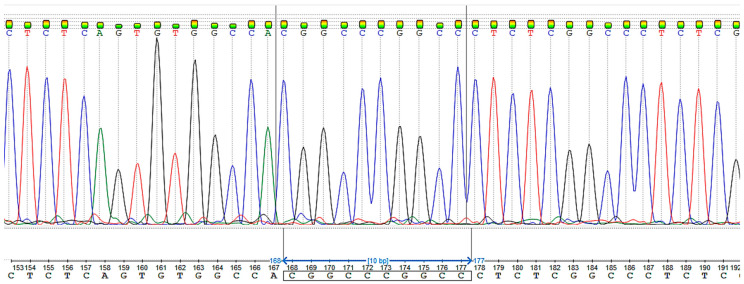
Patient Sanger sequencing. The duplication of five nucleotides–CGGCC.

Sanger sequencing confirmed the chr17:1,770,026A>ACGGCC mutation in the patient (Figure 2).

**Figure 3:**
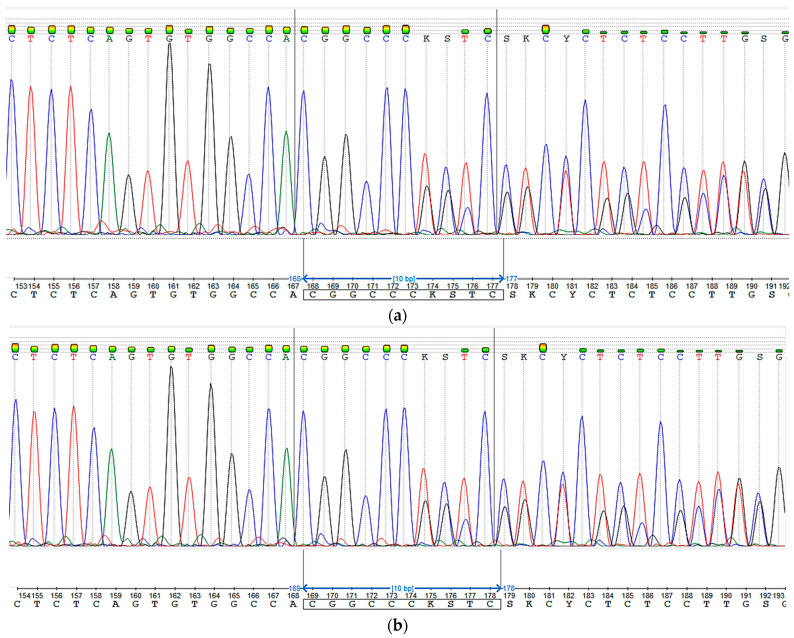
Sanger sequencing: c.259_260insCGGCC heterozygous mutation; (**a**) mother (**b**) father.

Sanger sequencing confirmed the chr17:1,770,026A>ACGGCC mutation in the patient’s parents (Figure 3). Both parents were found to be heterozygous for the mutation c.259_260insCGGCC.

### 3.3. Likelihood Ratio (LR) for Relative Pairs Using Autosomal STRs

Even though the parents of the proband claim no family ties, their carriage of an ultra-rare pathogenic variant indicates the possible presence of such a relationship. To test this hypothesis, we used a system of microsatellite markers to assess the degree of relationships between the proband’s mother and father. Based on multiplex PCR analysis, data were obtained for 21 autosomal highly polymorphic genomic DNA loci: D3S1358, TH01, D12S391, D5S818, TPOX, D2S441, D7S820, D13S317, FGA, D22S1045, D18S519, D18S519, CSF1PO, D6S1043, vWA, D21S11, SE33, D10S1248, D1S1656, D19S433, and D2S1338. To calculate the degree of parental relatedness, the obtained data were loaded into the Familias program. We compared allele sequences and corresponding putative allele lengths using autosomal STR markers. Next, we assessed the weight of the evidence for a certain relationship with likelihood ratio (LR), which involves a comparison of the probabilities of the DNA profiles under two alternative propositions (Hp and Hd) [4]. LRs < 1 imply that the genetic evidence indicates that the putative family member is less biologically related. A total of 124 Tuvans were analyzed, including the patient’s parents. Some of the results of the likelihood ratio (LR) and identical-by-state (IBS, the presence of the same nucleotide sequence) analysis are presented in Table 1.

## 4. Discussion

Type VI OI was first described in 2002 by Glorieux et al. (2002) [5]. The authors reported eight patients with a clinical manifestation atypical for the first four OI types. Patients with type VI OI were more likely to have fractures than patients of other known types, and the disease manifested from four months of age. All patients had vertebrae compression fractures and decreased bone mineral density; histology revealed a characteristic pattern of “fish scales”. The patients did not have dentin anomaly, and there was also no blue sclera. The molecular genetic analysis did not reveal significant mutations in the *COL1A1* and *COL1A2* genes. In 2011, Becker J. et al. described mutations in the *SERPINF1* gene as the cause of type VI OI. The authors identified a homozygous mutation in the *SERPINF1* gene in a sick proband whose parents are second cousins. Additionally, this mutation was detected in the sibling; this indicates an autosomal recessive type of inheritance [6]. Further studies also showed an autosomal recessive inheritance [7,8,9].

*SERPINF1* is on the short arm of chromosome 17 (17p13.3); it contains 9 exons and encodes a pigment epithelium-derived factor (PEDF). Protein product refers to neurotrophic proteins. PEDF has a high affinity for the collagen of the extracellular matrix. It was shown that PEDF plays a role in bone homeostasis as an inhibitor of bone resorption, and it inhibits the maturation of osteoclasts by blocking RANKL-mediated proliferation and the differentiation of osteoclast precursors [7,10]. It is also known that PEDF suppresses the expression of the gene that inhibits mineralization, which leads to an increase in osteoblast enzymes and an increase in matrix mineralization [11]. To date, according to the ClinVar database, 234 variants in the gene have been registered, 93 of which are associated with OI and 68 with type VI OI. Of these, 19 variants are pathogenic, 15 are likely pathogenic, and 17 have conflicting interpretations of pathogenicity. There are the following molecular consequences in genes: frameshift (13), missense (73), nonsense (13), splice site (4), and UTR (untranslated region) (67).

In our work, we described a patient with multiple fractures during the first year of life: a closed fracture of the right femur with displacement, a closed fracture of the tibial shaft in the lower third, and a closed fracture of the left humerus with displacement, a consolidated fracture of 8-9-10 ribs on the right, a consolidated combined compression fracture of the L1-L3 vertebrae, a closed fracture of the right humerus in the upper third without displacement, and a closed consolidating fracture of the right radius without displacement in the proximal section. The patient has no hearing impairment or blue sclera. The patient has muscle weakness; the boy is in a supine position. The patient was sent for target panel sequencing, including the genes *COL1A1* and *COL1A2*. We have not found pathogenic mutations in these genes. The next step was whole-exome sequencing. We found a novel homozygous mutation in the gene *SERPINF1*. Identifying a mutation in this gene makes it possible to give a diagnosis of VI OI, which is important for determining a treatment strategy. There are studies that show that, in addition to bisphosphonates, denosumab is used to treat VI OI [12].

In the present case, a homozygous mutation implies the inheritance of mutations from both parents or the occurrence of a de novo mutation. Sanger sequencing analysis showed a heterozygous mutation in both parents, which explains the occurrence of a homozygous mutation in a child. The proband’s parents deny family ties and claim that they come from different settlements. The degree of relationships analysis between the proband’s parents showed LR-2.26355, IBS = 2–15%, IBS = 1–55%, and IBS = 0–30%, which is consistent with the population sample of Tuvans and shows a low probability of related relationships, in comparison with values already known for individuals TKT150 and TKT151 who are parent and child and have rates of LR-50281500, IBS = 2–27.27%, IBS = 1–72.73%, IBS = 0–0%.

Tuvans belong to the native Siberian ethnic group. Tuvans are one of the most compactly living peoples of Russia, settled mainly in the territory of Tuva. The number of Tuvans is over 250 thousand people in Russia. They belong to the Mongoloid race and speak the Tuvan language, which belongs to the Turkic language family. The maximum closeness of the gene pool of the Tuvans with the Altaians, Khakas, and Shors is shown [13]. 

The frequency-analysis results of autosomal SNPs by various methods, the composition similarity of the Y-chromosome haplogroups, and YSTR haplotypes show that the gene pool of Tuvans is very heterogeneous in terms of the composition of genetic components. The majority of the most frequent Y-chromosome haplogroups in the Tuvans demonstrate the founder effect. Furthermore, for hereditary hearing loss, common haplotypes specific for recessive mutations *GJB2* c.516G>C, c.-23+1G>A, and c.235delC in the indigenous Turkic-speaking peoples of Siberia were identified, which confirms the decisive role of the founder effect in the high prevalence of these mutations [14]. The incidence of OI in the Russian Federation is 1.08 per 100,000 children, according to the 2015 Federal Register [15]. According to 1999 data, two patients with OI from the same family were registered [16]. There are no data on the molecular genetic features of OI in the Tuvan population. The mutation found in our study also may be ethnospecific for this region, which requires further research to determine the mutations causing OI in Tuvans.

## 5. Materials and Methods

### 5.1. Massively Parallel Sequencing and Sanger Sequencing

We used target panel sequencing for the analysis of mutations in the *COL1A1* and *COL1A2* genes, the enrichment method using long-range PCR. DNA libraries were prepared using the Nextera Flex kit (Illumina, San Diego, CA, USA) according to the protocol recommended by the manufacturer. The analysis was performed by next-generation sequencing on a MiSeq Sequencing System (Illumina, San Diego, CA, USA). We carried sequencing data processing out under GATK best practices for Germline SNPs Indels using the GATK4 software suite. We assessed the quality of readings using the Qualimap program. Alignment of reads to the full sequence of the *COL1A1* and *COL1A2* (GRCh38/hg38) genes was performed using BWA. The Annovar program was used for annotation.

To perform whole-exome sequencing, the patient’s DNA was analyzed on a new generation NextSeq 2000 Sequencing System (Illumina Inc., San Diego, CA, USA) with an average coverage of ×73. Targeted enrichment using the Agilent Sure Select All Exon v8 kit (Agilent, Santa Clara, CA, USA) was used for sample preparation. 

Sequencing data were analyzed using the GATK Best Practices Germline short-variant discovery pipeline. Reads were aligned to the reference human genome sequence (hg38), with the post-processing alignment, variant calling, quality filtering, and annotation of the identified variants by the canonical transcript of each gene. Variants that did not meet the quality criteria were excluded from further analysis. The pathogenicity of the variants was determined considering the ACMG recommendations. Reads had a length of 2 × 101 bp. The total reads 34,693,457 direct and the same number of reverse ones. The median reliability of nucleotide determination was higher than Q30. The median coverage was 65×, and the mean coverage was 72.8×.

Sanger sequencing around the identified variant was performed in both directions for a sample from the patient and the samples from his parents (for the establishment of a mutation carrier). The following primers for the PCR were selected: forward: CAAGACTTCCTGTCTCCTGCCA; reverse: TGCTACTTCACCCCTCGCT.

### 5.2. Calculating the Degree of Relationships of the Proband’s Parents

We used the microsatellite markers system of the COrDIS “EXPERT 26” kit to assess the degree of relationships of the studied DNA samples from the proband’s mother and father. The PCR products of individual loci were separated on a NANOFOR-05 genetic analyzer (Synthol) in the presence of DNA length standards S550 (CORDIS) under the conditions recommended by the manufacturer. We performed fragment-size analysis using GeneMarker Software (V.3.0.1) (State College, PA, USA). We used the Familias program to calculate the probability of a relationship and identification based on the DNA data [17]. A database of allele frequencies of 20 autosomal STRs was used. This database was created from a population sample of reference DNA profiles of Tuvans. 

To search for any possible combinations of DNA profiles with each other and estimate the likelihood ratio (LR), we used the “blind search” module: parent–child, siblings, half-siblings, cousins and second cousins, sisters, and direct matches. The competing hypothesis was that the samples were unrelated.

## 6. Conclusions

In conclusion, this report expands the list of pathogenic variants in the gene *SERPINF1* that cause type VI OI. We describe a new homozygous mutation in the *SERPINF1* gene in a Tuvan patient with multiple fractures, which is consistent with the diagnosis of osteogenesis imperfecta type VI. Our study demonstrates the usefulness of whole-exome sequencing in diagnosing groups of diseases with a common clinical picture, such as osteogenesis imperfecta, in order to establish a diagnosis and select the most appropriate therapy for the patient.

## Figures and Tables

**Table 1:** Likelihood ratio and identical-by-state analysis for relative pairs.

Person 1	Person 2	Relationship	LR	IBS = 2	IBS = 1	IBS = 0
TKT150	TKT151	Parent–child	50,281,500	27.27%	72.73%	0%
TKT150	TKT151	Siblings	4,092,930	27.27%	72.73%	0%
TKT72	TKT73	Siblings	271,411	40.91%	36.36%	22.73%
TKT150	TKT151	Cousins	2166.26	27.27%	72.73%	0%
TKT81	TKT141	Parent–child	317.711	4.55%	95.45%	0%
TKT18	TKT142	Cousins	2.26667	18.18%	45.45%	36.36%
TKT152	TKT178	Cousins	2.26382	9.09%	50%	40.91%
D6262	D6263	Cousins	2.26355	15%	55%	30%
TKT50	TKT143	Cousins	2.25966	9.09%	54.55%	36.36%
TKT68	TKT76	Cousins	2.2494	18.18%	40.91%	40.91%
TKT138	TKT182	Cousins	2.24883	18.18%	63.64%	18.18%
TKT171	TKT177	Second cousins	2.24573	4.55%	54.55%	40.91%
D6262	D6263	Second cousins	1.45411	15%	55%	30%
D6262	D6263	Half-siblings	1.1542	15%	55%	30%
D6262	D6263	Direct match	2.7 × 10^−16^	15%	55%	30%
D6262	D6263	Parent–child	0.0	15%	55%	30%

Note: LR—likelihood ratio, IBS—identical-by-state (total number possible shared for the overlapping markers): IBS = 0 (none overlapping markers), IBS = 1 (overlapping markers with 1 allele), IBS = 2 (overlapping markers with 2 alleles).

## Data Availability

Due to the privacy policy, data are available on request after approval of the patients.
